# UBASH3B promotes tamoxifen resistance and could be negatively regulated by ESR1

**DOI:** 10.18632/oncotarget.23608

**Published:** 2017-12-22

**Authors:** Ketao Jin, Huanrong Lan, Junyu Zhang, Jieqing Lv, Yuan Chen, Kang Yu, Wei Wang

**Affiliations:** ^1^ Department of Colorectal Surgery, Shaoxing People’s Hospital, Shaoxing Hospital of Zhejiang University, Shaoxing, 312000, Zhejiang Province, P.R. China; ^2^ Department of Breast and Thyroid Surgery, Shaoxing People’s Hospital, Shaoxing Hospital of Zhejiang University, Shaoxing, 312000, Zhejiang Province, P.R. China; ^3^ Department of Hematology, The Fifth Affiliated Hospital of Wenzhou Medical School, Lishui, 323000, Zhejiang Province, P.R. China; ^4^ Department of Pathology, Zhejiang Provincial People’s Hospital, Hangzhou, 310014, Zhejiang Province, P.R. China; ^5^ Department of Hematology, The First Affiliated Hospital of Wenzhou Medical School, Wenzhou, 323000, Zhejiang Province, P.R. China

**Keywords:** UBASH3B, breast cancer, tamoxifen resistance, TP53 mutation, prognosis

## Abstract

**Purpose:**

To explore the prognostic value of UBASH3B in ER+ breast cancer patients and explore potential molecular mechanisms.

**Materials and Methods:**

Datasets from The Cancer Genome Atlas (TCGA) and Gene Expression Omnibus (GEO) were re-analyzed to explore the association between UBASH3B and the progression of ER+ breast cancer. Kaplan-Meier plot analysis with a total of 734 ER+ samples and Gene Set Enrichment Analysis with 632 samples were used in the study.

**Results:**

High expression of UBASH3B is negatively correlated with distant metastasis free survival (DMFS, *P =* 0.01, *P =* 0.045, *P =* 0.04 in 2 independent datasets and a merged dataset, respectively), disease specific survival (DSS, *P =* 0.028) and disease free survival (DFS, *P =* 0.0052, *P =* 0.011, *P =* 0.016 in 3 independent datasets, respectively) in ER+ breast cancer patients. Subset analysis found that UBASH3B also has prognostic value on both lymph node positive and negative sub-populations with ER+ breast cancer. This study also demonstrates that UBASH3B expression is tightly associated with tamoxifen resistance and TP53 mutation, which explains the association between UBASH3B and poor prognosis of ER+ breast cancer. Further analyses show that the expression of UBASH3B is affected by promoter methylation and copy number loss. Besides, UBASH3B is inversely correlated with ER and down-regulated by ER. Importantly, we find cisplatin could be a therapeutic option targeting on UBASH3B in clinical settings.

**Conclusions:**

UBASH3B is negatively regulated by ER and confers poor outcome in ER+ breast cancer patients. Cisplatin is a potential therapeutic option for the management of breast cancer patients with high expression of UBASH3B.

## INTRODUCTION

Breast cancer is the most common malignancy and the leading cause of cancer death in females worldwide [1]. The number of cases has significantly increased since the 1970s, a phenomenon partly attributed to the wide use of menopausal hormone therapy and increased breast cancer screening [2]. It is estimated that there would be 231840 new cases and 40290 deaths of female breast cancer in the United States in the year 2015 [3].

Among all types breast cancer, over 70% are ER positive (ER+) according to molecular subtyping based on therapeutic regimens [4]. The selective ER modulator tamoxifen forms a central modality in the treatment of ER+ breast cancer, and demonstrates remarkable efficacy especially in patients with early breast cancer. However, approximately 40% patients with ER+ breast cancer are insensitive to tamoxifen treatment and even for those patients who show response initially would become refractory to tamoxifen-directed therapy ultimately, requiring physicians to consider how to better initiate the next step in therapy [4]. One of the key challenges Physicians facing today is to identify of patients best suited for and most likely to respond to each drug. Finding new predictive biomarkers and targets to enhance tamoxifen sensitivity and moreover reverse the resistant phenotype in ER+ breast cancer remain critical goals.

UBASH3B, also called Suppressor of T-Cell Receptor Signaling 1 (STS-1), could promote accumulation of epidermal growth factor receptor (EGFR) on the cell surface by inhibiting degradation of EGFR [5]. Recently, Lee et al. showed that UBASH3B was overexpressed in Triple Negative Breast Cancer (TNBC), where it supported cancer proliferation, invasion, and metastasis largely through up-regulation of epidermal growth factor receptor (EGFR). They also demonstrated that UBASH3B is a functional target of microRNA200a (miR200a) that is down-regulated in TNBC, which they thought could explain why UBASH3B is overexpressed in TNBC [6]. This is a great success in searching for potential therapeutic targets and management of TNBC. Nevertheless, they left several important questions unanswered.

The goal of the present study was to clarify the prognostic value of UBASH3B in ER+ breast cancer and elucidate its association with tamoxifen efficacy. We also sought to uncover new regulators of UBASH3B and search for available chemotherapeutics that could target UBASH3B and enhance tamoxifen sensitivity.

## RESULTS

### UBASH3B is associated with poor survival in ER+ breast cancer patients

**Figure 1:**
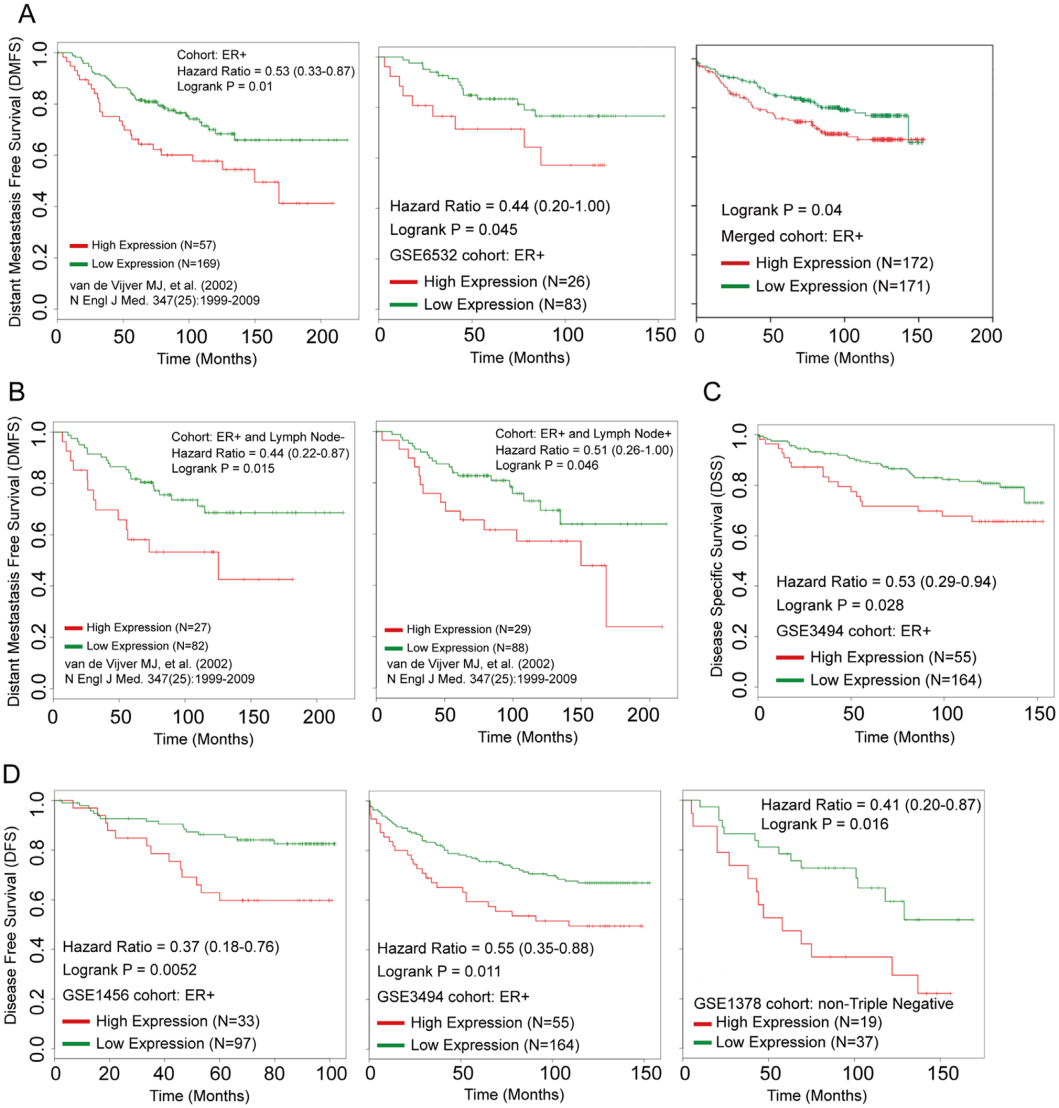
UBASH3B is associated with poor prognosis of ER+ breast cancer patients Kaplan-Meier plot of Distant Metastasis Free Survival (DMFS) in ER+ (**A**), ER+ breast cancer patients with lymph node negative and lymph node positive (**B**). (**C**) Kaplan-Meier plot of Disease Specific Survival (DSS) in ER+ breast cancer patients. (**D**) Kaplan-Meier plot of Disease Free Survival (DFS) in non-Triple negative breast cancer patients.

To deduce the potential role for UBASH3B in ER+ breast cancer, gene expression analysis based on publicly available datasets was performed. Interestingly, high expression of UBASH3B was closely associated Distant Metastasis Free Survival (DMFS) in Van dataset and GSE6532 (Figure 1A, left and middle graph, *P* = 0.01 and 0.045, respectively) and a merged dataset (Figure 1A, right graph, merged by GSE1456 and GSE3494, *P* = 0.04). Subset analysis indicated that UBASH3B expression was also correlated with DMFS of both lymph node – (Figure 1B, left, *P* = 0.015) and lymph node + (Figure 1B, right, *P* = 0.046) patients with ER+ breast cancer in Van dataset. These results indicate an association between UBASH3B and distant metastasis of ER+ breast cancer.

We also performed data analysis to assess the expression level of UBASH3B in relation to Disease Specific Survival (DSS) and Disease Free Survival (DFS). The results indicated that high expression of UBASH3B confers poor DSS in GSE3494 (Figure 1C, *P* = 0.028) and poor DFS in ER+ (Figure 1D, left and diddle: GSE1456 and GSE3494, *P* = 0.0052 and 0.011, respectively) or non-TNBC (Figure 1D, right: GSE1378, *P* = 0.016) cohorts. This observation suggests that high expression of UBASH3B may predict early death and disease progression in ER+ breast cancer patients.

Taken together, these findings suggest a potential role of UBASH3B in the progression of ER+ breast cancer.

### Overexpression of UBASH3B is correlated with tamoxifen resistance and TP53 mutation

**Figure 2:**
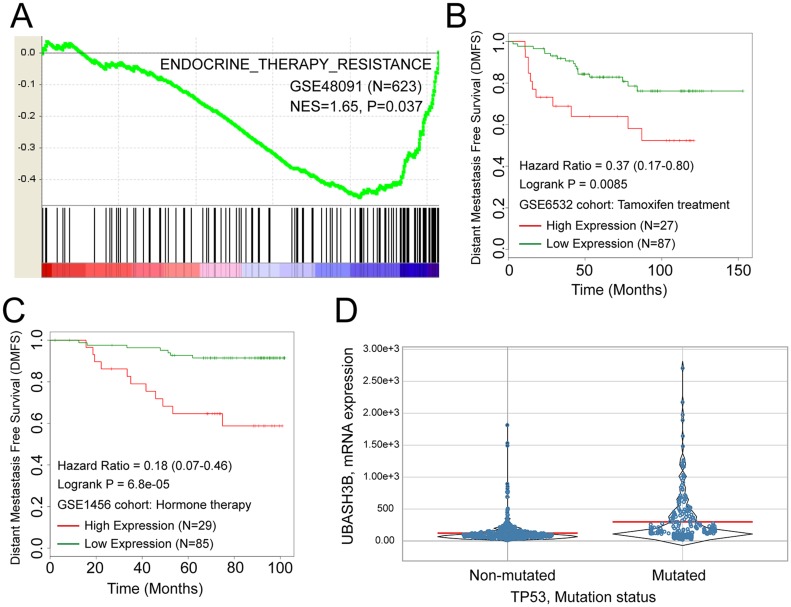
UBASH3B is negatively correlated with genes down-regulated in tamoxifen resistant patients (**A**) Kaplan-Meier plot of DMFS in breast cancer patient cohort received tamoxifen or hormone therapy (**B**, **C**). High expression of UBASH3B is associated with TP53 mutation in breast cancer (**D**).

Since high expression of UBASH3B confers poor prognosis in ER+ breast cancer and tamoxifen is a first-line drug for the management of ER+ patients, UBASH3B expression may be associated with tamoxifen efficacy. By performing GSEA using gene expression data from 623 breast cancer samples, we show high expression of UBASH3B is negatively correlated with genes down regulated in tamoxifen resistant patients, which indicates the association between UBASH3B and tamoxifen resistance (Figure 2A). Furthermore, high expression of UBASH3B was correlated with early distant metastasis in two independent cohorts (GSE6532 and GSE1456) that received tamoxifen treatment (Figure 2B and 2C, *P* = 0.0085 and 6.8e-05, respectively). Moreover, we found UBASH3B expression was higher in breast cancer patients with TP53 mutation compared with patients with wild type TP53, which may partly account for the poor prognosis in patients with high expression of UBASH3B (Figure 2D).

These results indicate that UBASH3B overexpression is correlated with tamoxifen resistance and TP53 mutation.

### Regulators of UBASH3B expression

**Figure 3:**
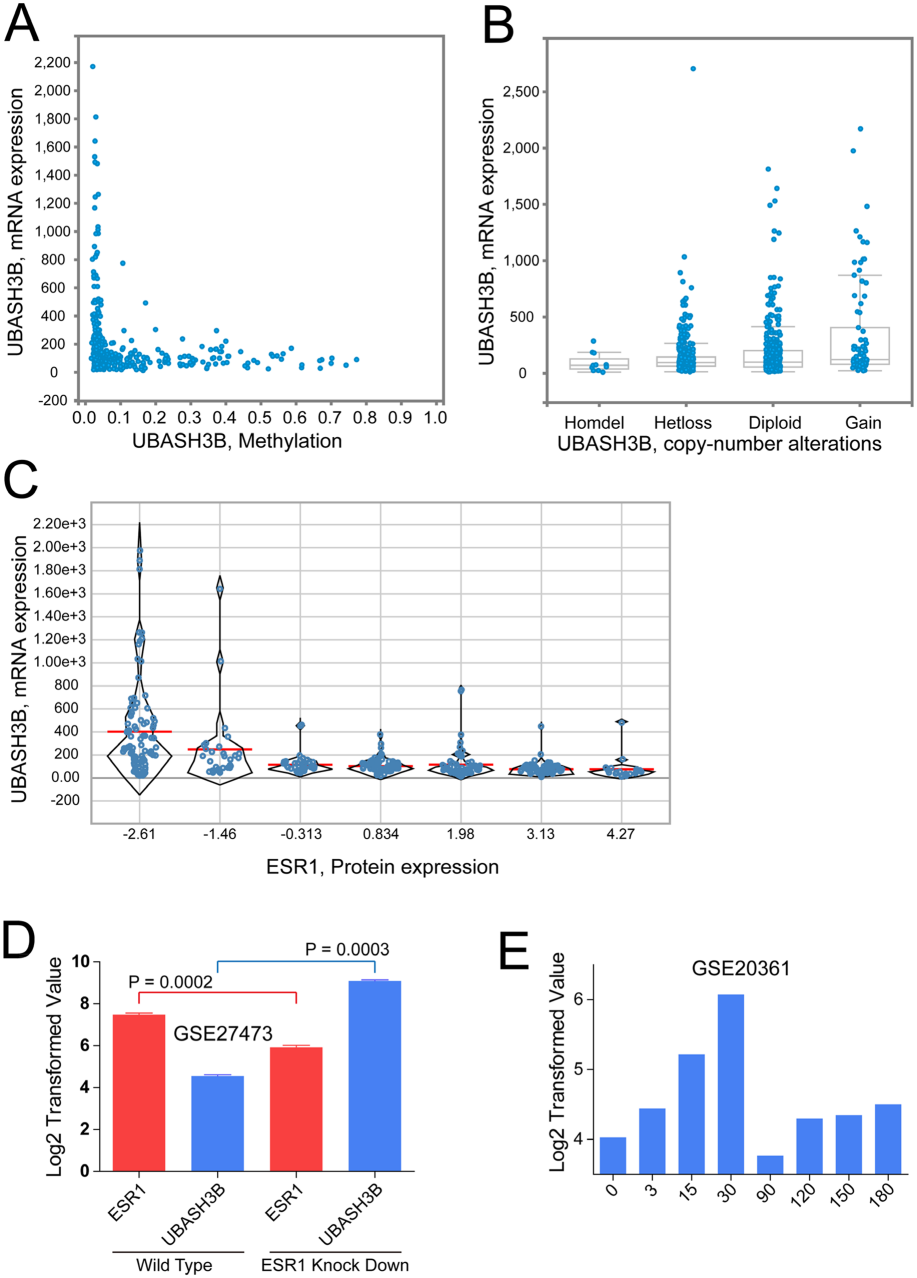
UBASH3B expression is negatively regulated by ESR Scatter plot of UBASH3B mRNA expression values against UBASH3B methylation levels (**A**), UBASH3B copy number alterations (**B**) and ESR1 protein expression values (**C**). (**D**) Log2 transformed mRNA expression values of UBASH3B and ESR1 in wild type and estrogen receptor (ER) knock down MCF-7 breast cancer cells. (**E**) Log2 transformed mRNA expression values of UBASH3B in MCF-7 cells after 3, 15, 30, 90, 120, 150 and 180 days of estrogen deprivation. *P* values were calculated by paired two-tailed *t* test. Error bars represent mean ± SEM.

Findings in previous sections show potential roles of UBASH3B plays in the progression and tamoxifen resistance in ER+ breast cancer. Next, we sought to the factors that could influence the expression of UBASH3B. Datasets from TCGA and GEO were used in the analyses. The results indicated that UBASH3B mRNA expression is inversely correlated with its methylation levels (Figure 3A) and copy number variation (Figure 3B). Interestingly, we found mRNA expression of UBASH3B is also negatively correlated with ESR1 protein levels (Figure 3C), thereby a critical question arises. Could ESR1 regulate the expression of UBASH3B? Here we show that UBASH3B mRNA expression is 16 fold higher in ER knock down MCF-7 cells than in parental controls (Figure 3D). Data also suggests that this negative regulation is associated with estrogen stimulation. In long term estrogen deprived conditions, UBASH3B expression in day 30 is about 4 fold higher compared to day 0 (Figure 3E). These observations show that ER is a crucial negative regulator of UBASH3B expression.

## DISCUSSION

Although tamoxifen has been successfully used in the management of patients with ER+ breast cancer, resistance is a common problem that ultimately culminates in treatment failure. Identification of predictive biomarker of tamoxifen resistance and targets to enhance its sensitivity is more and more important in the years to come.

UBASH3B contain an SH3 domains and could promote cancer progression by targeting CBL ubiquitin ligase for dephosphorylation and inactivation, which in turn leads to up-regulation and accumulation of activated EGFR [5]. Recently, Lee et al. reported that UBASH3B was overexpressed in TNBC and confers poor overall survival in patients with ER- breast cancer, but no significant disadvantage in patients with ER+ breast cancer [6]. In the present study, we demonstrate that high expression of UBASH3B is associated with poor prognosis of ER+ breast cancer and tamoxifen resistance. Furthermore, the expression of UBASH3B is inversely correlated with its methylation levels and copy number variation. Interestingly, our result also indicates that UBASH3B expression is negatively regulated by ER and this regulation is associated with estrogen stimulation. Since tamoxifen functions largely as agonist in molecular level and could recapitulate the gene expression profile induced in breast cancer cells by estrogen [18], here we hypothesized that UBASH3B expression is negatively regulated by tamoxifen through activating ER signaling pathway in tamoxifen sensitive patients while not in tamoxifen refractory patients, which can perfectly explain the correlation between UBASH3B expression and tamoxifen efficacy.

In an effort to find available chemotherapeutics that could be used in adjuvant setting for the management of breast cancer patients with high expression of UBASH3B, data mining from The Comparative Toxicogenomics Database (CTD) was performed [19]. Our result suggests that cisplatin is a potential therapeutic option that could down-regulate the expression of UBASH3B and enhance tamoxifen sensitivity.

**Figure 4:**
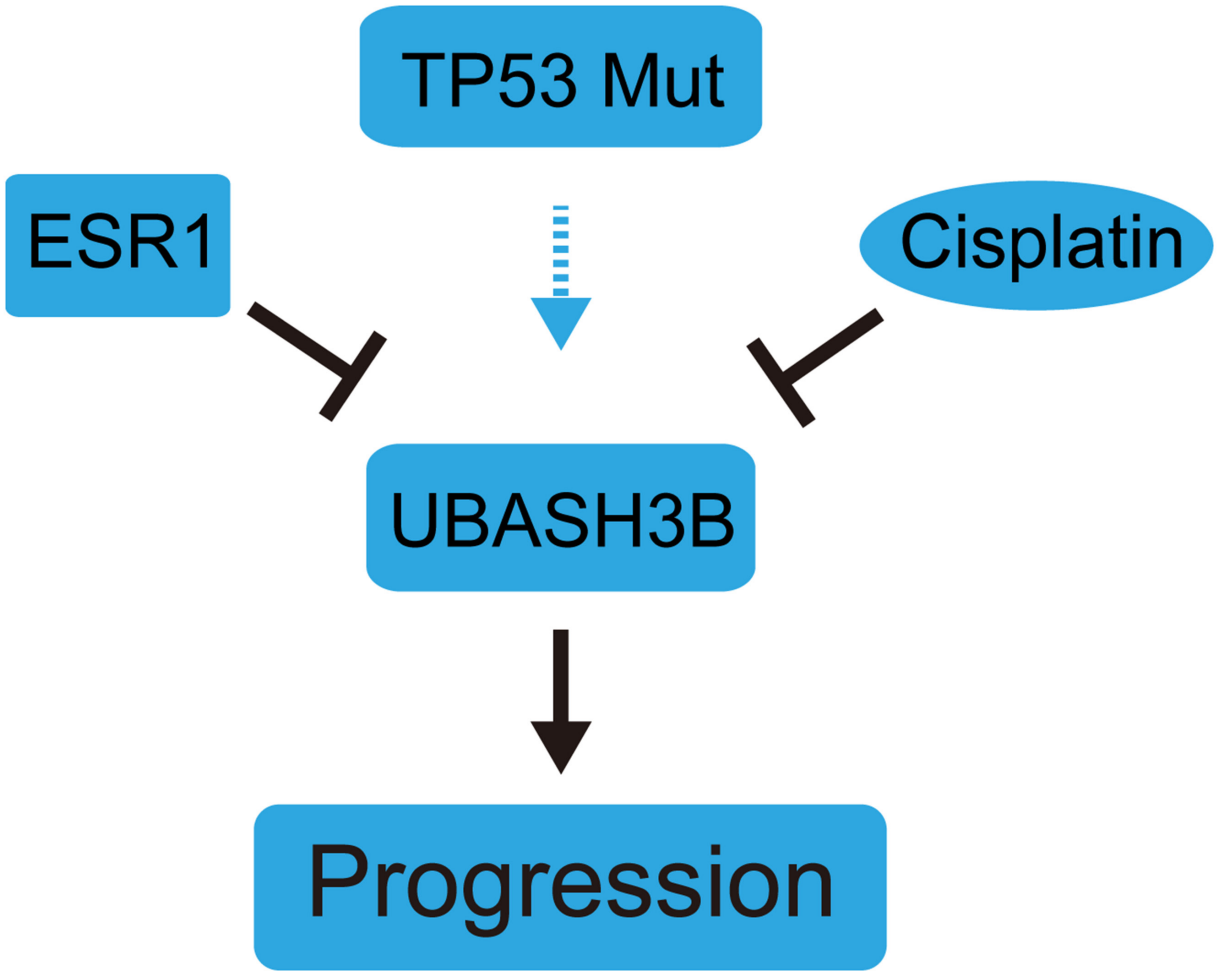
Diagram of UBASH3B signaling pathway This graph shows that UBASH3B is negatively regulated by ER and Cisplatin and could promote breast cancer progression. TP53 mutation may also promote breast cancer progression by regulating UBASH3B expression.

Finally, the overview of UBASH3B signaling pathway was demonstrated in Figure 4. This diagram shows that UBASH3B is down-regulated by ER and cisplatin and can promote breast cancer progression through EGFR. The effect of UBASH3B on cancer progression may be enhanced by the co-existence of TP53 mutation.

Taken together, we suggest that UBASH3B expression levels could be used for patient stratification and directing personalized therapies. Cisplatin is a potential therapeutic option that may be used in combination with tamoxifen to enhance the sensitivity of tamoxifen. Nevertheless, *in vivo* and *in vitro* experiments and randomized controlled clinical trials are still needed.

## MATERIALS AND METHODS

### Ethics statement

We are free to use breast cancer data in the cancer genome atlas (TCGA) by meeting its freedom-to-publish criteria: A marker paper has been published on that tumor type. We have the right to use datasets from Gene Expression Omnibus (GEO) by complying with all requirements according to each dataset. The Research Ethics Committee of Zhejiang Provincial People’s Hospital waived the requirement for ethical approval of this analysis because the registry is a de-identified database. Written consent was obtained from all alive patients.

### Genomic analysis

UBASH3B mRNA expression, methylation and copy number alteration (CNA) data were obtained from the TCGA Data Portal (National Cancer Institute, The cancer genome atlas data portal, https://tcga-data.nci.nih.gov/docs/publications/tcga/, Accessed September 1, 2013). All other datasets are downloaded from NCBI GEO database [7]. TCGA data was used to demonstrate the association between UBASH3B mRNA expression and methylation or CNAs. The relation among UBASH3B expression and TP53 mutation status were also analyzed using TCGA data [8]. Van dataset [9], GSE6532 [10], GSE1456 [11], GSE3494 [12] and GSE1378 [13] were used for survival analysis. GSE27473 [14] and GSE20361 [15] were used to show the negative regulation of UBASH3B by ER. Gene Set Enrichment Analysis (GSEA) [16] was performed using expression profiles of 632 breast cancer samples (GSE48091) [17]. The mRNA expression profiling of all the samples were performed on the Human U133B Gene Chip and Human genome U133 plus 2.0 platforms (Affymetrix, Santa Clara, CA).

### Statistical analysis

Standard statistical tests were used to analyze the clinical and gene expression profiling data, including the log rank test, fisher exact test and independent samples *t*-test. Significance was defined as a *P* value of less than 0.05. Analyses were primarily performed using R 3.0.1 (R Foundation for Statistical Computing [http://www.r-project.org/]), GraphPadPrism5.01 (GraphPad Software, Inc. [www.graphpad.com]) and SPSS version21 (SPSS Inc, Chicago, Illinois).
